# Electrospin-Coating of Paper: A Natural Extracellular Matrix Inspired Design of Scaffold

**DOI:** 10.3390/polym11040650

**Published:** 2019-04-09

**Authors:** Kelvin Ng, Pedram Azari, Hui Yin Nam, Feng Xu, Belinda Pingguan-Murphy

**Affiliations:** 1 Department of Biomedical Engineering, Faculty of Engineering, University of Malaya, Kuala Lumpur 50603, Malaysia; ngkelvin88@hotmail.com (K.N.); pedram.azari@gmail.com (P.A.); 2 Centre for Applied Biomechanics, Department of Biomedical Engineering, Faculty of Engineering, University of Malaya, Kuala Lumpur 50603, Malaysia; 3 Tissue Engineering Group, Department of Orthopaedic Surgery (NOCERAL), Faculty of Medicine, University of Malaya, Kuala Lumpur 50603, Malaysia; huiyin26@yahoo.com; 4 The Key Laboratory of Biomedical Information Engineering of Ministry of Education, School of Life Science and Technology, Xi’an Jiaotong University, Xi’an 710049, China; fengxu@xjtu.edu.cn; 5 Bioinspired Engineering and Biomechanics Center (BEBC), Xi’an Jiaotong University, Xi’an 710049, China

**Keywords:** electrospinning, tissue engineering, paper-based scaffolds, osteoblast proliferation, polycaprolactone

## Abstract

Paper has recently found widespread applications in biomedical fields, especially as an alternative scaffolding material for cell cultures, owing to properties such as its fibrous nature, porosity and flexibility. However, paper on its own is not an optimal material for cell cultures as it lacks adhesion moieties specific to mammalian cells, and modifications such as hydrogel integration and chemical vapor deposition are necessary to make it a favorable scaffolding material. The present study focuses on modification of filter paper through electrospin-coating and dip-coating with polycaprolactone (PCL), a promising biomaterial in tissue engineering. Morphological analysis, evaluation of cell viability, alkaline phosphatase (ALP) activity and live/dead assays were conducted to study the potential of the modified paper-based scaffold. The results were compared to filter paper (FP) and electrospun PCL (ES-PCL) as reference samples. The results indicate that electrospin-coating paper is a simple and efficient way of modifying FP. It not only improves the morphology of the deposited electrospun layer through reduction of the fiber diameter by nearly 75%, but also greatly reduces the scaffold fabrication time compared to ES-PCL. The biochemical assays (Resazurin and ALP) indicate that electrospin-coated filter paper (ES-PCL/FP) provides significantly higher readings compared to all other groups. The live/dead results also show improved cell-distribution and cell-scaffold attachment all over the ES-PCL/FP.

## 1. Introduction

Paper, with history dating back thousands of years to when it was initially invented as a tool to record and preserve information, has recently found widespread applications in biomedical fields, such as paper-based electronics [1], low-cost and disposable analytical platforms [2,3,4], microfluidic devices for drug screening [5], and disease modeling [6,7,8,9]. As a biomaterial, paper offers several advantages such as low cost of production, biocompatibility, ease of chemical alteration, and physical modifiability [10]. For example, paper has shown great potential as an alternative scaffolding material for cell cultures owing to properties such as its fibrous nature, porosity and flexibility. Its porous structure produces a wicking capability which allows the flow of medium across the paper, thus facilitating the transportation of nutrients and release of waste products [11]. The cellulose fibers in the paper provide a platform for physical and biological support of the cultured cells, enabling them to grow into functioning tissue in an in vitro environment [10,12,13,14]. These advantages exhibited by versatile paper materials have enticed researchers into further exploring the potential of the paper as a cell culture scaffold. 

Although paper has several favorable properties for biomedical applications, on its own, it is not an optimal material for cell cultures. Some properties, such as lack of cell adhesion moiety [15] and relatively large pore size, have been found to create difficulty for cell migration along the paper scaffold, in which the cell will need to take a longer path in situation where the void gap is too wide for the filopodia to bridge [10]. Several studies have shown that modification of paper is necessary to manipulate its chemical and physical properties and create a more feasible environment for cell proliferation, migration, and differentiation [16,17]. Various modification methods for paper have been reported, including binding of ceramics to the paper surface with latex binder to modify the topography [18], chemical vapor deposition of polymers onto paper to increase the cell adhesion moiety for better cell growth [9], wax printing on paper to manipulate the proliferation direction of cells, and the addition of hydrogels to permit better cell migration within a stacked-up paper scaffold [7,8]. Most of these modification methods use polymers to modify the properties of paper scaffolds to be more biochemically favorable for cell culture. While for scaffolding applications it is critically important to maintain the porous structure of paper upon the addition of polymer [19], some of the modification methods involve use of heat or chemicals that might damage paper’s natural fibrous microstructure. Furthermore, the pore size and fiber diameter of paper are far bigger than natural extracellular matrices (ECM), which is an interwoven random fibrous structure primarily made of proteins (mainly collagen and elastin), proteoglycans, and glycosaminoglycans [20,21]. ECM plays a crucial role in providing support for cell attachment and growth as well as serving as a reservoir of water, nutrients, cytokines, and growth factors for maintenance of tissue homeostasis [20]. An ideal scaffold must mimic the structure and geometry of ECM to significantly enhance tissue formation [13] and provide explicit biophysical and biochemical cues to distinctly modulate cellular response for promoting tissue restoration.

The electrospinning technique has gained popularity in recent years as a feasible method to fabricate biomimetic polymeric scaffolds that resemble native ECM [22,23,24,25]. Among a wide range of polymers that have been electrospun [26], polycaprolactone (PCL) has found extensive applications in tissue engineering. PCL is soluble in organic solvents, such as chloroform, and toluene at room temperature with low cost of synthesis [27]. When PCL is electrospun, apart from its favorable intrinsic properties, it also gains advantages such as high surface-area-to-volume ratio, high porosity and an interconnected porous network, all of which are crucial in a biomimetic material [28]. However, the hydrophobic nature of PCL is not favorable for cell adhesion as it prevents cells from proliferating into the pores of the scaffold, leading to low cell affinity and uncontrolled biological interactions with the scaffold [27,29]. Additional surface modification methods are often required to alter the surface hydrophobicity for the improvement of cell affinity [30,31]. Blending or using PCL in combination with other hydrophilic biopolymers has been a very popular strategy to address some of the shortcomings of PCL [29]. In a recent study, Yew et al. reported modification of nitrocellulose membranes with electrospun PCL, where electrospun PCL was able to alter the wicking properties of the membrane and enhance the protein binding, as well as improving detection sensitivity of nucleic acid-based lateral flow assay [28]. Protein binding is an important aspect of scaffolds for cell anchoring and a combination of paper and PCL has the potential to produce a scaffold with a controllable degree of hydrophilicity and wicking properties. Further, electrospinning PCL on paper could resolve the morphological issue of paper for cell work and produce an ECM-like scaffold, with paper performing as a reservoir basement layer to enhance the nutrient delivery. However, the potential for modification of paper-based scaffolds with electrospun PCL has not yet been explored.

Bone tissue lesions—one of the most widely occurring injuries to human body—are results of trauma, increased life expectancy, ageing population and obesity. It is estimated that bone injuries requiring treatment will double between 2012 and 2020 [32]. Tissue engineering-inspired therapies are the only feasible solution to the current clinical challenge of providing bone replacements [33]. Bone cells (osteoblasts, osteocytes and osteoclasts), as the building blocks of bone tissue, have increasingly been the subject of studies in vivo and in vitro due to their significant role in acceleration of bone tissue regeneration. The need for improved scaffolding materials to function as an artificial ECM, to facilitate the localization and delivery cells to the desired sites in the body, is never ending [34]. Paper-based platforms have shown great potential in osteogenic differentiation of stem cells [9,35]. The promising results obtained in previous studies motivated us to use human fetal osteoblast (hFOB) to evaluate the in vitro performance of our scaffold designs. Although translation of in vivo results into practice is easier, in vitro studies provide a more cost-effective initial insight in terms of cell viability and scaffolds capability in maintaining phenotype and functionality [36]. Besides, progress in understanding the causes of in vivo transplantation failure has led to improvements in design of scaffolding materials to provide more accurate results in relevance to future transplantation trials [36,37].

This article focuses on the modification of paper with PCL and studies scaffolds in terms of physical and morphological properties, as well as hFOB cell viability. To evaluate the effect of a combination of the filter paper (FP) and PCL, two groups of samples were prepared through electrospin-coating and dip-coating. FP and electrospun PCL (ES-PCL) were used as reference samples. The results suggest that electrospin-coating of paper has great potential to improve cell viability and tissue formation as a scaffolding material. 

## 2. Materials and Methods 

### 2.1. Dip-Coating of Filter Paper (DFP)

**Figure 1:**
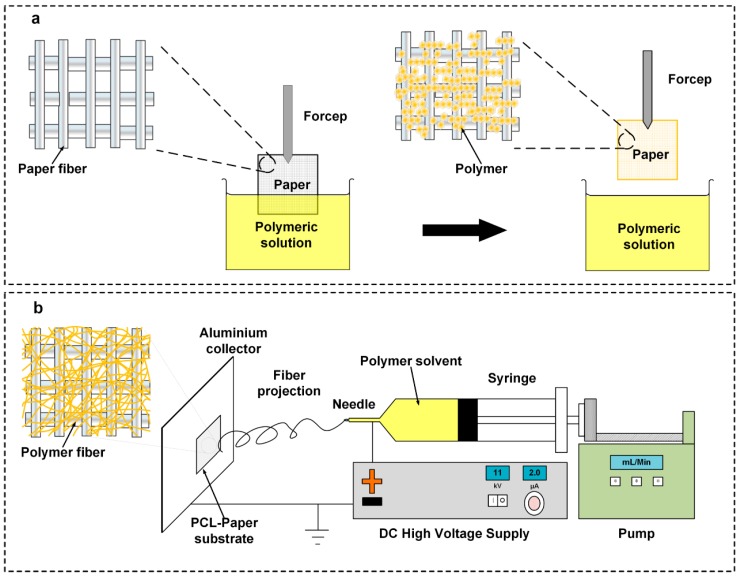
Schematics of filter paper modification. (**a**) Dip-coating of filter paper with polycaprolactone (PCL) solution (DFP), (**b**) Electrospinning of PCL on Filter paper (ES-PCL/FP).

The filter papers (Whatman^®^grade 114) were cut into 1 × 1 cm pieces using a paper guillotine cutter. The cut filter paper was submerged in a 10 wt.% solution of PCL (Sigma Aldrich, St. Louis, MO, USA, Mn = 80,000 g/mol) in chloroform (Frinedemann Schmidt, Perth, Australia) for approximately 3 s and then left to dry at room temperature (Figure 1a).

### 2.2. Electrospin-Coating of Paper (ES-PCL/FP)

The spinning dope was prepared by dissolving PCL pellets (10% w/v) in a co-solvent mixture, comprising 9 volumetric parts of chloroform and 1 volumetric part of N, N-dimethylformamide (DMF) (Sigma Aldrich, St. Louis, MO, USA). The solution was prepared at room temperature under magnetic stirring in a condensed conical flask to obtain a clear homogenous solution. The solution was loaded into a 10 mL syringe (Terumo) with a 20 G blunt needle horizontally placed 18 cm from the aluminum collector. The feeding rate of the syringe pump (KD-100, KD Scientific Inc, Holliston, MA, USA) was adjusted at 3 mL/h. The electrospin-coating was carried out using a DC voltage of 12 kV (Gamma High Voltage Research, Ormond Beach, FL, USA) while a piece of filter paper (5 cm × 5 cm) was stuck on the aluminum collector (Figure 1b). The duration of the electrospin-coating was 30 min.

### 2.3. Electrospun PCL (ES-PCL)

Electrospinning of PCL was carried using the same parameters reported in Section 2.2 directly on aluminum foil. 20 mL PCL solution was electrospun to obtain a scaffold with sufficient thickness for cell culturing.

### 2.4. Porosity Measurement 

The porosity of the scaffolds was measured using a Pycnometer (Marienfeld, Lauda-Königshofen, Germany) based on the Archimedes Principle. Absolute ethanol (John Kollin Corporation, Midlothian, UK) was used as the medium and gravimetric displacement of liquid was measured at room temperature [38]. The porosity of the scaffolds was calculated based on Equation (1).

(1) $$\mathrm{Porosity}\;\left(\%\right)=\frac{W_{2}-W_{3}-W_{m}}{\rho_{e}}/\frac{W_{1}-W_{3}}{\rho_{e}}\times 100$$

where *W*_1_ represents the weight of the pycnometer filled with absolute ethanol, *W*_2_ is the weight of pycnometer filled with absolute ethanol and scaffold, *W*_3_ is the weight of pycnometer and absolute ethanol when the ethanol-soaked scaffold had been taken out from *W*_2_, *W*_m_ is the dry weight of the scaffold, and ρ_e_ is the density of the absolute ethanol.

### 2.5. Characterization of Scaffold Mechanical Properties 

Tensile tests were carried out using an INSTRON 3345 device with a load of 100 N based on ASTM D 638. The specimens were punched in dumbbell-shaped geometries with dimensions of 50 mm × 9 mm (length x width). The support span length was set at 50 mm. The testing speed was set at 1 mm/min. The strain (ε) was evaluated as the ratio between the elongation of the specimen Δl and the original length l_0_ as shown in Equation (2).

ε = Δl/l_0_ (2)

### 2.6. Field Emission Scanning Electron Microscopy (FESEM)

The morphology of the scaffolds was studied using FESEM (Carl Zeiss Auriga, Oberkochen, Germany). Samples were sputter coated with gold and analyzed under FESEM. For samples seeded with cells, cell fixation was conducted prior to coating as described in Section 2.8.4. The fiber diameter and pore size were measured based on the obtained FESEM images using ImageJ software (version 1.48).

### 2.7. Cell Culture

The osteoblast cell lines, hFOB 1.19, (ATCC, Manassas, VA, USA), were used in this research. hFOB cells were cultured in T-75 flasks (Thermo Fisher Scientific, Waltham, MA, USA) by using Dulbecco’s Modified Eagle’s Medium (DMEM F12, Gibco™, Waltham, MA, USA) with 10% fetal bovine serum (FBS, Gibco™, Waltham, MA, USA), and 1% Geneticin^®^ (G418, Thermo Fisher, Waltham, MA, USA). The cells were cultured at 34 °C in a humidified (5% CO_2_, 95% air) atmosphere. When cells reached confluence, cells were detached with Accutase^®^(Innovative Cell Technologies, USA). Cell suspension at a density of 5 × 10^6^ cells/ml was prepared for further use. 10 µL of the cell suspension was seed onto each presoaked scaffold (1 × 1 cm). The scaffolds were incubated for 2 hours. 1 mL of medium was micropipette into each well to submerge the scaffolds. Only passage 5 cells were used in this study.

### 2.8. Characterization of Cell Viability and Morphology 

#### 2.8.1. Resazurin Reduction Assay

The Resazurin Reduction assay was performed at three time points, namely, days 1, 4, and 7. Resazurin Stock solution was prepared by dissolving 140 mg of Resazurin powder (Sigma-Aldrich, St. Louis, MO, USA) into 1000 mL of phosphate buffer saline (PBS) (Sigma-Aldrich, St. Louis, MO, USA). Resazurin working solution was prepared by mixing stock solution with PBS at a volumetric ratio of 1:9. The samples were incubated for 4 h at 34 °C with a 5% CO_2_ atmosphere for the conversion of resazurin to resorufin. Triplicates of 100 mL of the Resazurin working solution incubated with cells grown on each scaffold were taken for absorbance measurements. The optical densities were then measured using a microplate reader (FLUOstar OPTIMA, BMG labtech, GmbH, Ortenberg, Germany) at an absorbance wavelength of 570 nm, with 595 nm set as the reference wavelength. Unseeded sterilized scaffolds were incubated under the same conditions in resazurin working solution as blanks and absorbance values were calculated accordingly.

#### 2.8.2. Live/Dead Confocal

The Live/Dead Viability/Cytotoxicity Kit (Life Technologies, Carlsbad, CA, USA) measures the cell viability based on the integrity of cell membranes. Specimens were supplemented with 10 μL of PBS mixture containing calcein-AM and ethidium homodimer-1 in a 1:4 ratio and incubated for 20 min at room temperature. 

#### 2.8.3. Alkaline Phosphatase (ALP) Assay

RIPA Lysis, extraction buffer and p-nitrophenyl phosphate (p-NPP) (Thermo Fisher, Waltham, MA, USA) were used to perform the ALP assay. pNPP solution used to lyse the substrate was incubated with cell lysate. ALP in cell lysate converted pNPP substrate to p-nitrophenol. A standard curve of Optical Density (OD) plotted against 4-nitrophenol concentration was generated and used in estimation of ALP activity. By comparing the OD obtained from reaction mixture to standard curve of 4-nitrophenol, the quantity of pNPP substrate converted to p-nitrophenol over time was estimated.

#### 2.8.4. Cell Fixation for FESEM

The samples seeded with cells were washed 3 times in PBS and fixed with 2.4% glutaraldehyde solution (Sigma Aldrich Co., St. Louis, MO, USA) for 24 h. After fixing, the specimens were washed 3 times again with PBS, placed through a series of graded ethanol solutions for dehydration, and allowed to dry overnight in a freeze dryer (FreeZone, Labconco, Hampton, NH, USA) prior to FESEM.

### 2.9. Statistical Analysis

For statistical analysis of biochemical assay results, a minimum of six technical replications (N = 6) were used for each group of samples. Data were tested by one-way analysis of variance (ANOVA) with Turkey post hoc test using IBM SPSS version 23 software (SPSS Inc., Chicago, IL, USA). P-values less than 0.05 (p < 0.05) were reported as statistically significant. 

## 3. Results and Discussion

### 3.1. Scaffold Morphological Analysis

**Figure 2:**
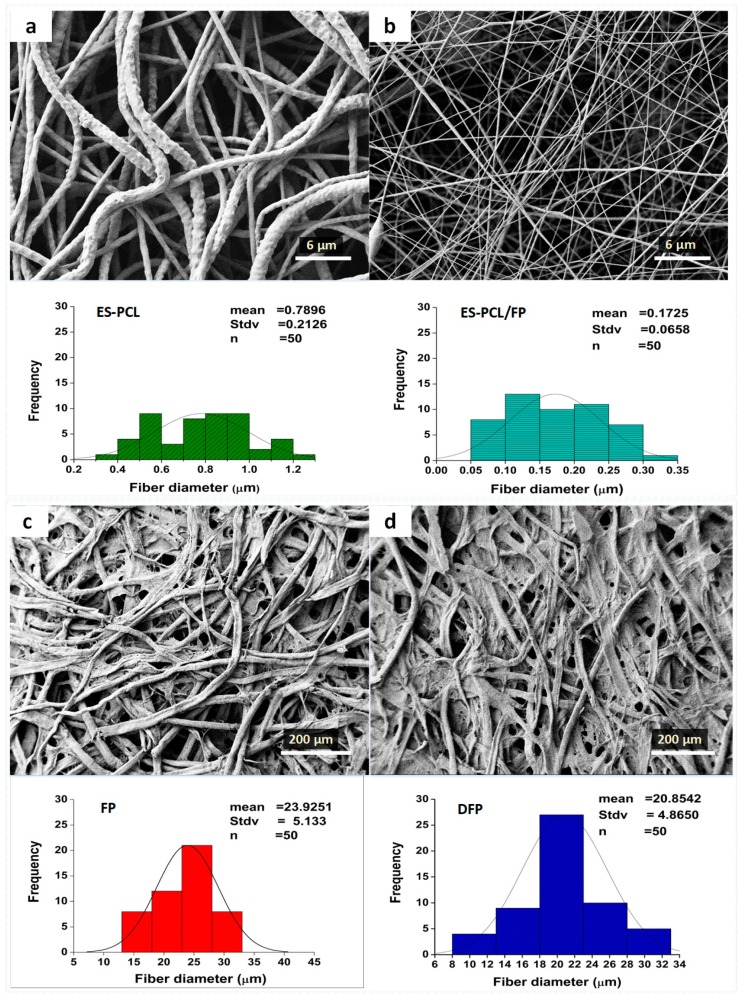
**Scaffold morphological analysis.** Field emission scanning electron microscopy (FESEM) microscopy of (**a**) ES-PCL, (**b**) ES-PCL/FP, (**c**) filter paper (FP) and (**d**) dip-coated filter paper (DFP) of their relevant fiber diameter distribution pattern.

Figure 2 shows the morphologies of 4 different types of scaffold, namely electrospun PCL (ES-PCL) (Figure 2a), electrospun PCL on filter paper (ES-PCL-FP) (Figure 2b), filter paper (FP) (Figure 2c) and dip-coated filter paper (DFP) (Figure 2d). With FP serving as the collecting platform during electrospinning, the diameters of the PCL fibers produced have been reduced by approximately 75% (Figure 1b). Separately, when FP was just dipped into the PCL solution, there was only a small change in FP fiber diameter, a reduction of 12%. Using filter paper as the collecting platform helps the removal of solvent from the electrospinning jet, through adsorption of excess solvent and facilitation of evaporation. Other studies reported that a higher rate of solvent removal leads to formation of fibers with smaller diameters. For example, Liu et al. reported that the porosity of the collector can affect the morphology of the deposited fibers [39]. Porous surfaces such as paper can affect the packing density of the deposited nanofibers as well as faster evaporation of residual solvents due to their higher surface area. Paper has the capability of absorbing liquid through capillary action, owing to its porous structure, which is able to absorb the residual solvent from the deposited fiber spun on the paper [10]. Wannatong et al. also reported that high boiling point solvents such as DMF, when used in electrospinning, could result in the formation of wet fibers on the collector [40], and that an increase in the evaporation rate of the solvent improved the morphology. Whatman 114# filter paper used in this research was capable of rapid absorption and enhancing the solvent evaporation. 

Table 1. shows the average porosity, pore size, thickness and tensile strength of the 4 types of scaffold. ES-PCL had the highest porosity (66.71%), followed by ES-PCL/FP (25.26%), then FP (7.55%) and the lowest was DFP (3.23%). The comparison of ES-PCL/FP and FP showed that coating a thin layer of PCL could increase the overall porosity of the scaffold more than twofold. However, the formation of thinner and straighter fibers on the surface of ES-PCL/FP, as indicated in Figure 1b, has resulted in a reduction in pore size. This was due to multiple layers of thin and straight nanofibers overlapping each other and leading to a reduction in the pore size. DFP had lower porosity compared to FP as PCL solution had further reduced its porosity to 50%. Besides, the more densely packed and overlapped nanofibers also increase the degree of fiber to fiber fusion, which could lead to a significant increase in tensile strength [41,42]. ES-PCL has the lowest tensile strength, while ES-PCL/FP with the smallest fiber diameters has the highest tensile strength (Table 1). However, a similar event was not observed in DFP, as non-homogenous distribution of PCL within FP does not contribute significantly to the tensile strength. In fact, electrospinning caused more PCL to be deposited on the FP (49.69 ± 9.3 µm thickness n = 7, (ES-PCL/FP-FP)) in comparison with the dipping method (37.78 ± 22 µm thickness n = 7, (DFP-FP)). This shows that electrospinning forms an integrated layer of PCL onto FP and the elastic nature of ES-PCL contributes to tensile properties. The microscopic interfacial view of the ES-PCL/FP is presented in supplementary material Figure S1.

### 3.2. Cell-Scaffold Interactions

**Figure 3:**
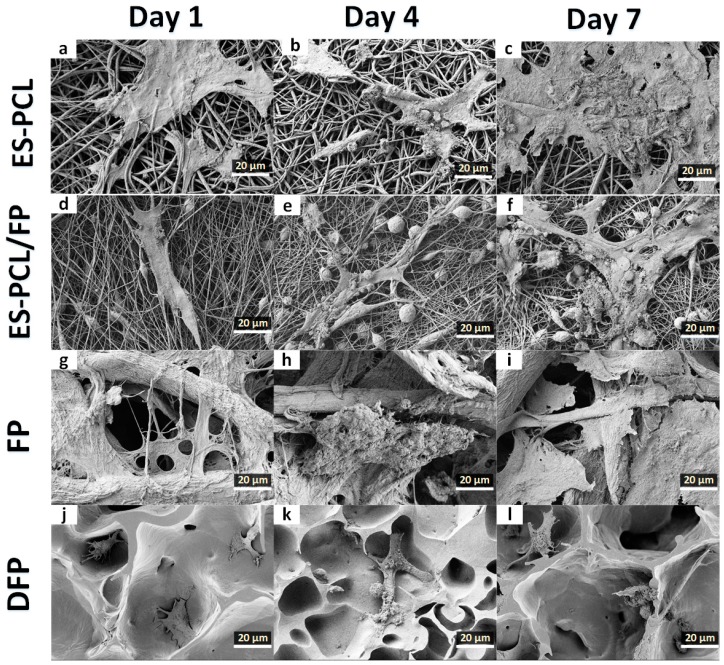
Cell morphological analysis. FESEM images showing the morphology of human fetal osteoblast (hFOB) plotted separately for each individual scaffold of ES-PCL (**a**–**c**), ES-PCL/FP (**d**–**f**), FP (**g**–**i**), and DFP (**j**–**l**) on different days. The magnification and resolution of all images are the same.

FESEM was used to evaluate morphology, adhesion, and the interaction of hFOB cells within the scaffold. Figure 2 shows the microscopic images of the hFOB cells on the scaffold on day 1, day 4 and day 7. For the ES-PCL scaffold (Figure 3a–c), the cells spread on the surface of the fiber and were more flattened, thicker and showed less distinct filopodia. As for the ES-PCL/FP scaffolds (Figure 3d–f), cells were observed to be more elongated, well-anchored to the fiber through distinct filopodia and microvilli and proliferating well along the direction of fiber alignment. In the FP scaffold (Figure 3g–i), the cells were observed to attach to the struts of cellulose fibers. Figure 3g shows that the cells bridged between two struts, whereas Figure 3h–i shows that the cells grew and covered up pores of the cellulose. As for the DFP scaffold (Figure 3j–l), the cells were seen mainly at areas where there are pores. The smooth surface and low porosity of the DFP scaffold (Figure 3j–l) does not provide a good surface for cells to attach and spread. Cells attached very poorly on the flat surface and with short filopodia. Furthermore, the reduced porosity of DFP may have prohibited cell penetration and infiltration within the scaffold. The micrograph clearly shows that the cell morphology and proliferation depend on the topography of the scaffold, such as fiber diameter, orientation and pore size [23,43].

### 3.3. Cell Proliferation Assay

**Figure 4:**
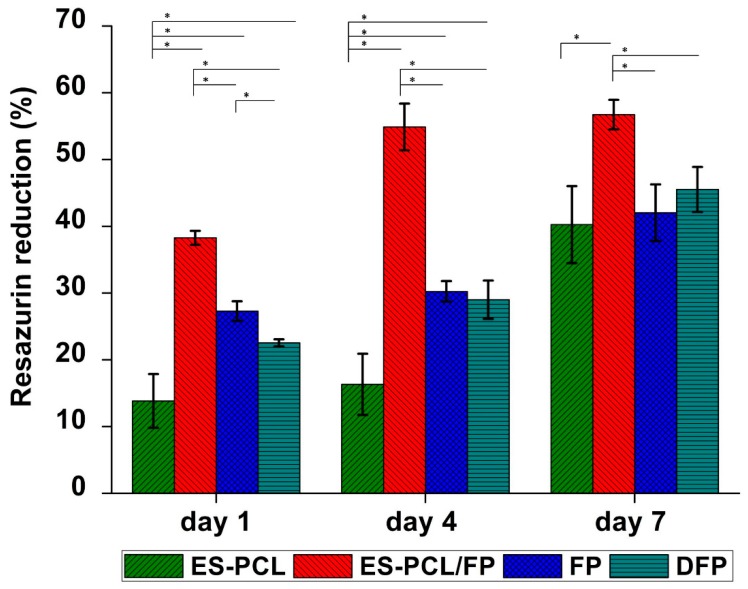
Cell proliferation analysis. Resazurin proliferation assay results for (ES-PCL, ES-PCL/FP, FP, and DFP). The resazurin reduction of hFOB increased on ES-PCL, ES-PCL/FP, FP, and DFP as a function of time. * indicates statistical significance (P < 0.05).

Figure 4 shows the metabolic activity of hFOB measured at the different time points (i.e., day 1, day 4 and day 7) for all groups of scaffolds. Comparison between the groups showed that ES-PCL/FP provided the highest proliferation readings on all days and the difference compared to other groups on the same day was significant. ES-PCL showed significantly lower metabolic activity on days 1 and 4 compared to other groups on the same day but reached the same level as FP and DFP on day 7. FP and DFP only indicated significant metabolic activity difference on day 1. While the two groups’ metabolic activity continued to rise on days 4 and 7, the difference was insignificant. 

As shown in Figure 4, the metabolic activity of different scaffolds ascended steadily from day 1 to day 7. The increase in metabolic activity for FP and DFP was significant throughout the time period. However, metabolic activity measured on ES-PCL/FP scaffolds showed significant increase only until day 4, and there was no significant difference between day 4 and day 7. This suggests that the cells on ES-PCL/FP may have reached confluency between the two time points. For ES-PCL scaffold, the metabolic activity on day 1 and day 4 was not significantly different, however there was a sharp increase in metabolic activity after day 4 and there was a difference in metabolic activity between day 4 and 7. This data is in line with the live/dead images, in which the cell density was almost the same on day 1 as on day 4.

### 3.4. Live/Dead Assay

**Figure 5:**
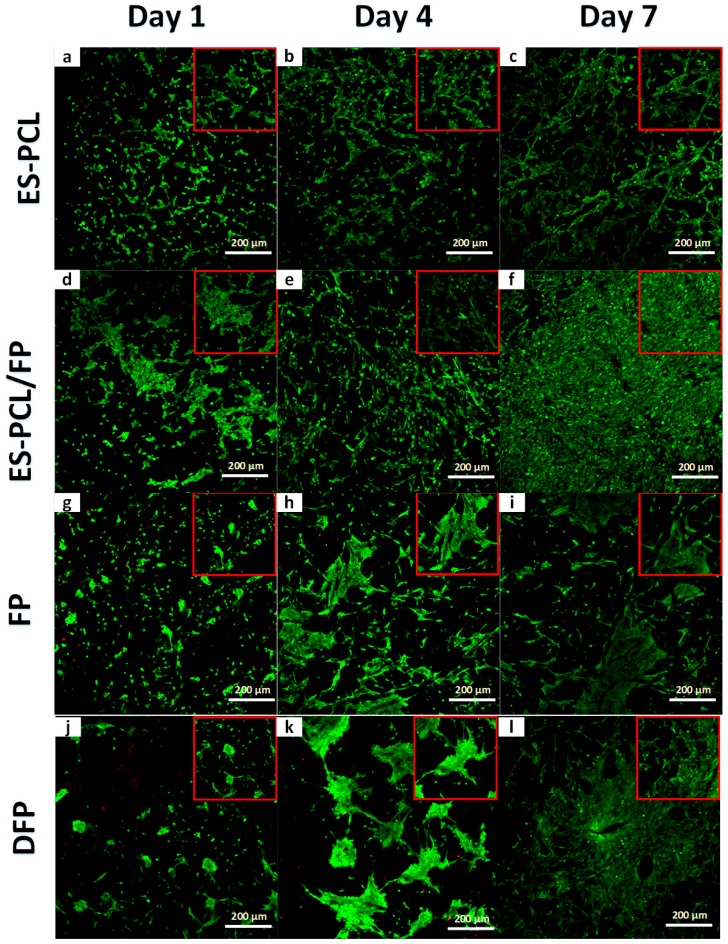
Cell viability analysis. Live/dead confocal of hFOB plotted separately for each individual substrate ES-PCL (**a**–**c**), ES-PCL/FP (**d**–**f**), FP (**g**–**i**), and DFP (**j**–**l**) on different days. The magnification and resolution of all images are the same.

Figure 5 shows the microscopic images of the scaffolds under fluorescent excitation using live/dead staining. Abundance of green spots indicates the high viability of hFOB cells on the scaffolds in a period of 7-day incubation. The confocal fluorescent excitation of blank scaffolds is presented in supplementary material Figure S2. As expected, the results suggest that paper and PCL are biocompatible substrates for cell culture. The abundance of green cells could suggest attachment of hFOB cells on the scaffolds as the images exhibited cells anchored on the surface with extended filopodia, a typical characteristic of osteoblast morphology [44]. There were clearly more live cells on the ES-PCL/FP scaffold in all three-time points (Figure 5d–f), cells were more well spread, and cell density was higher compared to other scaffolds on day 7 (Figure 5f). This result is in line with resazurin metabolic activity. The image (Figure 5f) also showed that the ES-PCL/FP surface was confluent with live cells without visible dead cells. DFP and FP scaffolds showed more individual cells. By day 4 cells on both DFP and FP scaffolds have spread out and occupied more space. The live-dead assay revealed that cell distribution was better on scaffolds with smaller fiber diameter than larger fiber diameters, as reflected by day 7 images (Figure 5c,f,i,l). Topography factors such as fiber diameter of a scaffold affect the cell growth, as the scaffold with a smaller fiber size provides a higher surface area for cell anchoring which leads to both higher cell growth and more even distribution [23,43].

### 3.5. Alkaline Phosphatase (ALP) Activity

**Figure 6:**
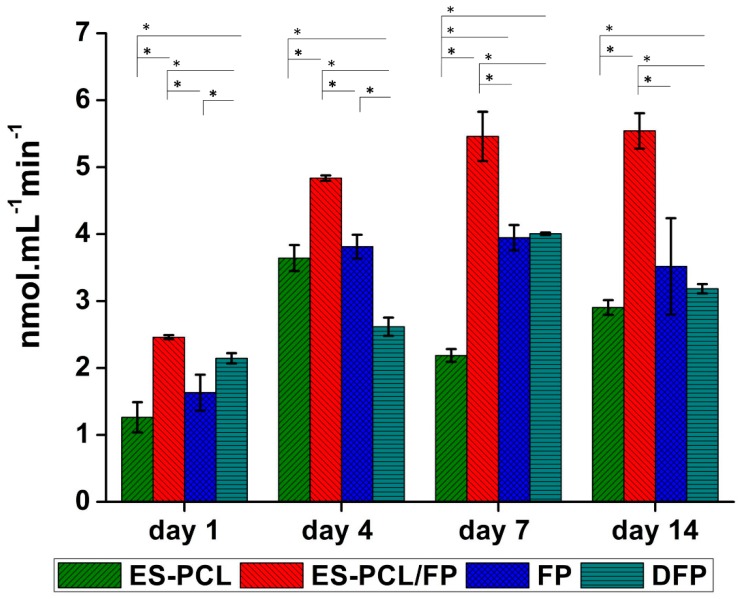
Bone metabolic activity analysis. Alkaline phosphatase (ALP) assay results for hFOB seeded-in (ES-PCL, ES-PCL/FP, FP, and DFP) at different time point. The ALP activity of hFOB increased on ES-PCL, ES-PCL/FP, FP, and DFP as a function of time. The results are statistically significant (P < 0.05).

Figure 6 shows the ALP activity of hFOB measured over a period of 14 days. As shown in Figure 6, the ALP activity on ES-PCL, FP and DFP scaffolds showed an ascending trend from day 1 till day 7. On day 14, DFP and -FP scaffolds showed a slight decrease in ALP activity. The ALP activity of ES-PCL/FP is significantly higher than other groups on all days. The ALP activity of ES-PCL/FP between day 7 and day 14 did not changed significantly, which indicates that the cells reached confluency, resulting in the ALP activity staying the same. The ALP activity results are in line with the resazurin assay, in which the ES-PCL/FP scaffold showed the highest readings in both. The ALP activity results also support the live/dead results. It has been reported that better distribution of cells can improve osteospecific differentiation [45]; based on live/dead assay (Figure 5f), ES-PCL/FP indicates the highest number of cells and cell spreading all over the surface of the scaffold. 

## 4. Conclusions

The present study evaluated the modification of FP through combination with PCL using electrospinning and dip coating methods, and their characteristics were compared to each other. The potential of the fabricated scaffolds in terms of cell viability (e.g., proliferation, adhesion and osteoblast cell activity (ALP)) was measured and compared. Dip coating of FP provided the least favorable cellular response within the groups. This was a result of reduced porosity of DFP, which decreased the number of anchoring spots for the cells and limiting the overall penetration and distribution of the cells within the scaffold. Dip coating of FP is similar to modification of paper using hydrogels, where in both methods polymer is applied to bridge the gap between paper pores to attain better cell attachment and distribution over the scaffold [6,7]. However, the hydrogels should have an edge over the dip coating with high molecular weight polymers as they do not obstruct cell penetration and distribution within the scaffold. While DFP and ES-PCL/FP used the same material combination, the comparison between the two groups showed that ES-PCL/FP is superior in cell viability and mechanical properties. This was due to better surface morphology and higher porosity of ES-PCL/FP. The biochemical assay results suggested that electrospin-coating of FP was a simple and effective way in which PCL and paper could complement each other. The fiber diameter of the electrospun PCL reduced greatly when it was collected on paper (ES-PCL/FP). The tensile strength of the ES-PCL/FP increased due to deposition of a layer of relatively elastic ES-PCL on top of FP and the rise in degree of fusion point. ES-PCL/FP also showed the highest metabolic and ALP activity as the most cell-viable among the scaffolds tested. The morphology of the cell attachment and distribution over the scaffolds indicated attachment to scaffold and anchoring of cells on ES-PCL/FP was more favorable. In conclusion, the electrospinning of PCL fibers on paper gives a synergistic outcome, in which the wicking capability of paper can retain cell suspension within the scaffold, improving the efficiency of cell seeding. Furthermore, electrospin-coating of paper reduces the scaffold fabrication time more than 10-fold compared to scaffolds fabricated purely based on electrospinning.

## Figures and Tables

**Table 1:** Porosity percentage and thickness of scaffold.

Scaffold Properties	ES-PCL	ES-PCl/FP	FP	DFP
**Tensile Strength (MPa)**	1.68 ± 0.23	5.80 ± 0.32	3.52 ± 0.38	3.54 ± 0.17
**Porosity (%)**	66.71 ± 2.65	25.26 ± 1.60	7.55 ± 2.76	3.23 ± 0.59
**Pore size (µm)**	13.11 ± 1.07	5.42 ± 0.22	92.13 ± 11.98	73.67 ± 17.00
**Thickness (µm)**	232.07 ± 11.59	258.78 ± 9.03	209.09 ± 0.50	246.87 ± 21.81

## Supplementary Materials

The following are available online at https://www.mdpi.com/2073-4360/11/4/650/s1. Figure S1. Optical Microscopy of ES-PCL/FP after 14 days of immersion in culturing medium. Figure S2. Live/dead confocal microscopy of blank scaffolds.
