# Supplementary material for: Electrospin-Coating of Paper: A Natural Extracellular Matrix Inspired Design of Scaffold
Source: Polymers (Basel). 2019 Apr 9;11(4):650. doi: 10.3390/polym11040650 (PMC6523310; doi:10.3390/polym11040650)
Supplement: Supplementary file 1 [file polymers-11-00650-s001.pdf]

polymers

MDPI

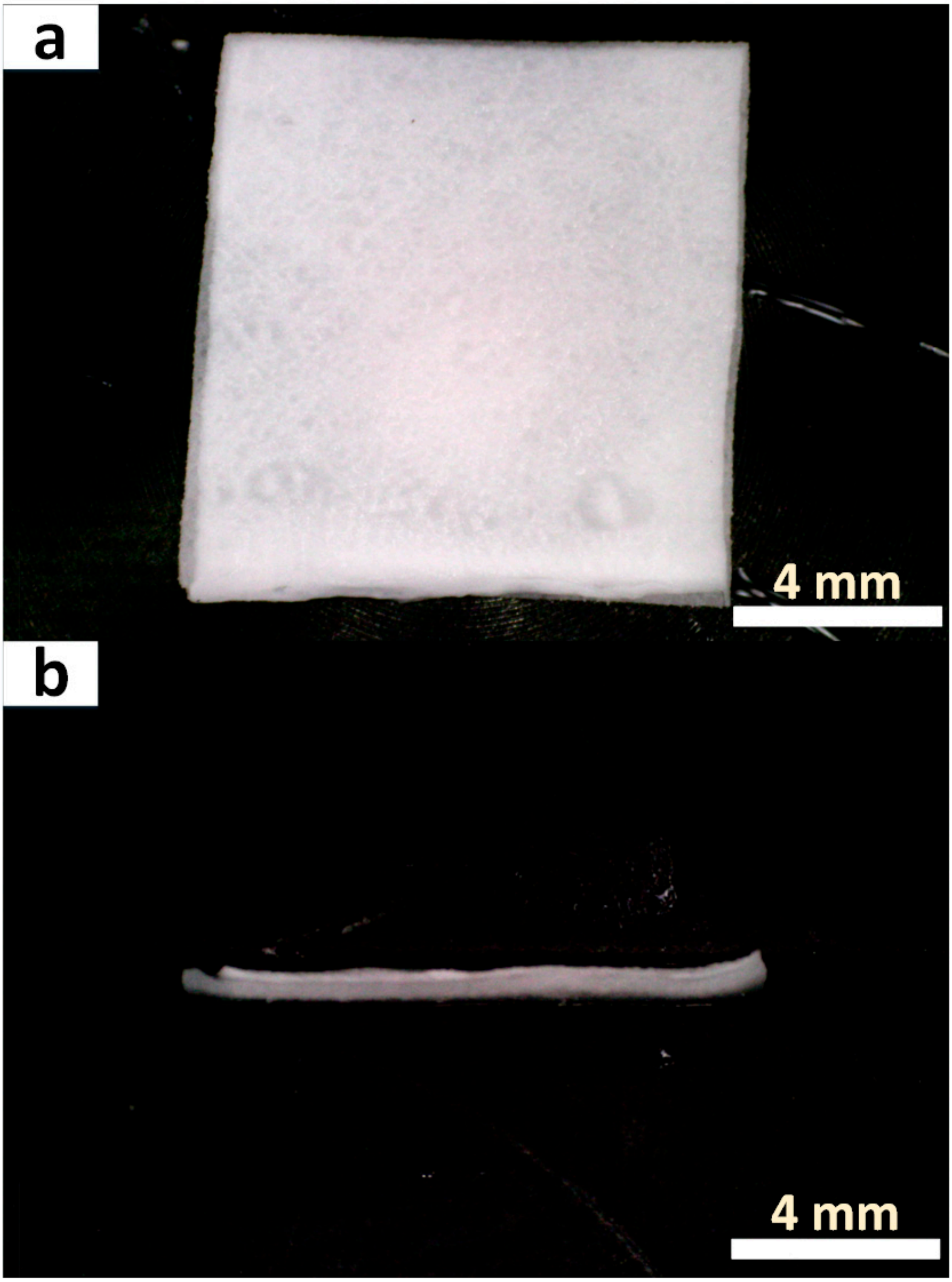

Figure S1. Optical Microscopy of ES-PCL/FP after 14 days of immersion in culturing medium. (a) Top view, (b) Side view

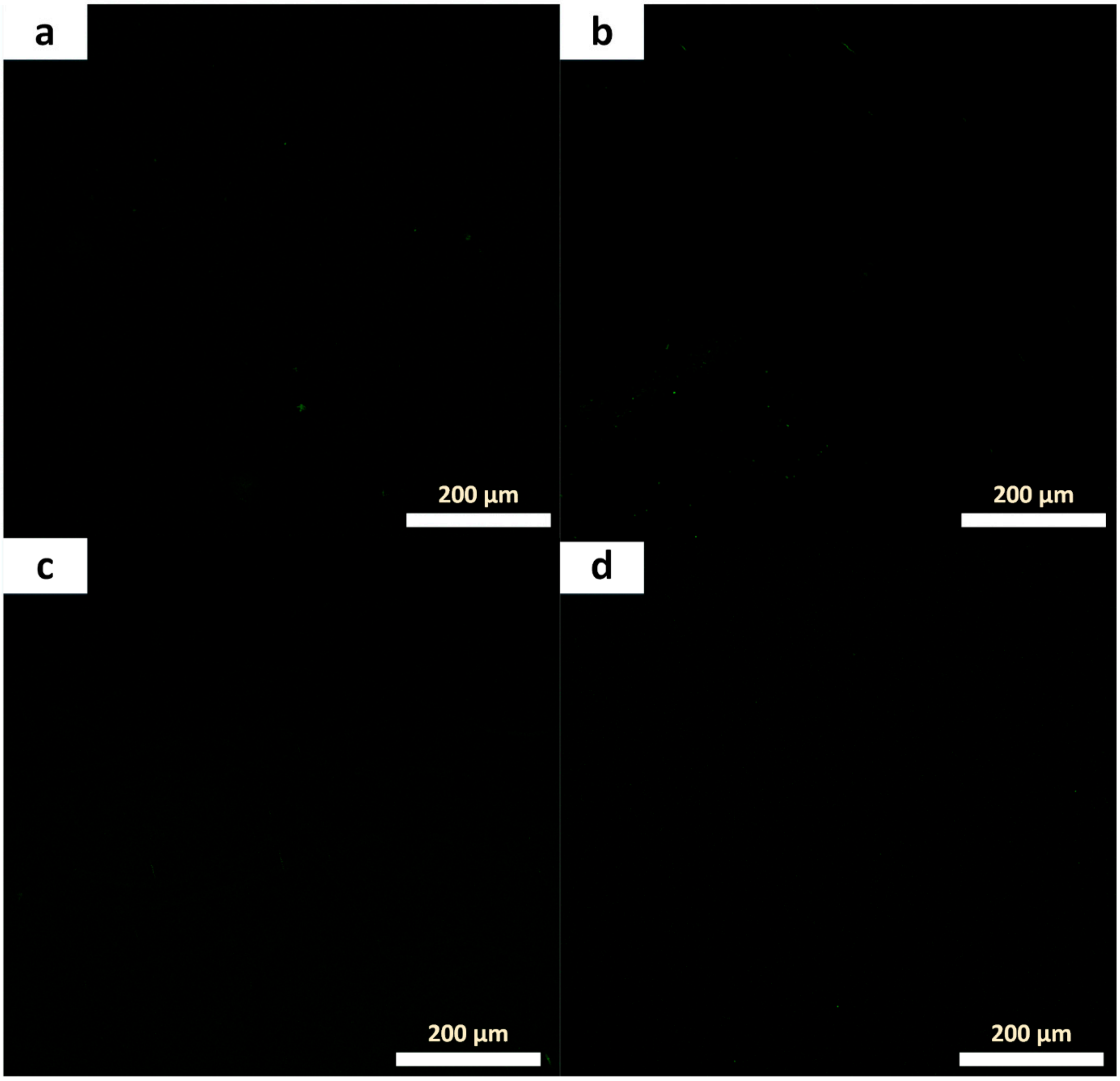

Figure S2. Live/dead confocal of blank scaffolds. (a) ES-PCL, (b) ES-PCL/FP, (c) FP, (d) DFP
